# Effects of the Barbell Load on the Acceleration Phase during the Snatch in Elite Olympic Weightlifting

**DOI:** 10.3390/sports8050059

**Published:** 2020-05-08

**Authors:** Ingo Sandau, Urs Granacher

**Affiliations:** 1 Research Group Weightlifting, Institute for Applied Training Science, D-04109 Leipzig, Germany; 2 Division of Training and Movement Sciences, University of Potsdam, Research Focus Cognition Sciences, D-14469 Potsdam, Germany; urs.granacher@uni-potsdam.de

**Keywords:** biomechanics, barbell velocity, performance, training, load–velocity relationship

## Abstract

The load-depended loss of vertical barbell velocity at the end of the acceleration phase limits the maximum weight that can be lifted. Thus, the purpose of this study was to analyze how increased barbell loads affect the vertical barbell velocity in the sub-phases of the acceleration phase during the snatch. It was hypothesized that the load-dependent velocity loss at the end of the acceleration phase is primarily associated with a velocity loss during the 1st pull. For this purpose, 14 male elite weightlifters lifted seven load-stages from 70–100% of their personal best in the snatch. The load–velocity relationship was calculated using linear regression analysis to determine the velocity loss at 1st pull, transition, and 2nd pull. A group mean data contrast analysis revealed the highest load-dependent velocity loss for the 1st pull (*t* = 1.85, *p* = 0.044, g = 0.49 [−0.05, 1.04]) which confirmed our study hypothesis. In contrast to the group mean data, the individual athlete showed a unique response to increased loads during the acceleration sub-phases of the snatch. With the proposed method, individualized training recommendations on exercise selection and loading schemes can be derived to specifically improve the sub-phases of the snatch acceleration phase. Furthermore, the results highlight the importance of single-subject assessment when working with elite athletes in Olympic weightlifting.

## 1. Introduction

In weightlifting, winning the competition means lifting the highest load in a particular bodyweight category. For this purpose, the athlete has to accelerate the barbell to a vertical velocity that is high enough to drop under the barbell and to catch it. Böttcher and Deutscher [1] defined this velocity as a “threshold velocity”, which is individual to every athlete and determines the minimal vertical velocity necessary for a successful lift. In other words, the highest weight an athlete can snatch is partially limited by his or her “threshold velocity”. From this perspective, the acceleration phase represents a prerequisite for a good lift. 

The acceleration phase during the snatch (i.e., pull) can be subdivided into three phases: 1st pull, transition, and 2nd pull [2], based on knee joint angle [3], vertical barbell velocity [4], or a combination of the two factors [1]. In the snatch, the velocity at the end of the 1st pull is, on average, around 1.06 to 1.50 m/s [5,6], and for the 2nd pull, 1.67 to 1.96 m/s [2,7,8]. During the transition, the velocity from the end of the 1st pull should be maintained or increased [9]. A loss in vertical velocity during the transition phase is deemed as a technical flaw that will result in lower snatch performance [9,10]. From a biomechanical standpoint, the vertical barbell velocity at the end of the 2nd pull (maximum vertical velocity) corresponds to the sum of the impulses (i.e., Δv) of each sub-phase of the entire acceleration phase.

It is evident that barbell-velocity and barbell-load share an inverse relationship known as the load–velocity relationship (LvR) [11,12]. With regards to weightlifting, the barbell load can be increased until the “threshold velocity” is reached. With increasing loads, a barbell velocity has to be achieved during each sub-phase (i.e., accelerative impulse) to realize the respective “threshold velocity” at the end of the 2nd pull. This ensures that subsequent movement phases (i.e., turnover, catch) can be executed successfully. The main goal during training with advanced weightlifters is to improve their strength capabilities [13,14] in order to provide an increased overall force output during the acceleration phase but not to maximize barbell acceleration. As a positive result of training, the load–velocity relationship should be shifted so that the “threshold velocity” is reached at a higher load. For this purpose, training of elite weightlifters consists of many exercises (e.g., snatch pulls, hang pulls, power snatch) and loading conditions to enhance the force output during the acceleration phase [15,16,17,18]. Unfortunately, the selection of exercise and loading condition for training the acceleration phase often depends on the coaches’ opinion rather than data from performance assessment.

As mentioned previously, each of the three sub-phases during the acceleration phase contributes to the maximal velocity at the end of the 2nd pull. A decrease in velocity during the single sub-phase influences the velocity at the end of the acceleration phase and the maximal load that can be lifted. Many studies exist on the biomechanical structure of the snatch during competition [2,6,8,19]. However, these studies did not address how increased barbell loads affect the biomechanics of the barbell during the acceleration phase. With regard to the snatch, only two studies are available that addressed this particular issue. When performing the snatch, increased barbell loads lead to decreased maximal vertical barbell velocity at the end of the 2nd pull (e.g., load–velocity relationship) [20,21]. Furthermore, results from Hadi, Akkuş, and Harbili [20] revealed that the increased load on the barbell produces the greatest effects on biomechanical measures such as time span, vertical work, and vertical power during the 1st pull. For the clean/clean pull and the snatch pull, Medvedjev et al. [22] and Frolov et al. [23] showed that with increasing loads there is an increase in time span for the 1st pull. In addition, analyses of the snatch during competition indicated that the 1st pull accounted for approximately 70% of the peak barbell velocity and that it spans approximately 65% of the entire time during the acceleration phase [6,24].

Taken together, it can be hypothesized that increases in the barbell load may affect the barbell velocity in sub-phases of the acceleration phase, which could have an impact on the maximal barbell velocity at the end of the 2nd pull. Within these sub-phases, it seems that an increase in barbell load will mainly affect the 1st pull in contrast to the transition or the 2nd pull. As a result, the 1st pull appears to have a large effect on the maximal velocity at the end of the 2nd pull and, therefore, on maximal snatch performance.

Thus, the purpose of this study was to investigate how the barbell load influences the loss of velocity during the 1st pull, transition, and the 2nd pull while performing the snatch in elite weightlifters. We hypothesized that a load-dependent decrease in velocity at the end of the 2nd pull is associated with a larger velocity decrease during the 1st pull compared with that experienced in the transition phase or the 2nd pull.

## 2. Materials and Methods

### 2.1. Participations

In this study, 14 male elite weightlifters aged 23.7 ± 3.2 years (body mass: 89.2 ± 23.5 kg, 1RM snatch: 152.9 ± 20.3 kg, 1RM clean and jerk: 185.9 ± 23.7 kg) from the German national team served as the experimental group. The bodyweight categories of the athletes were −62 kg (1 athlete), −69 kg (3 athletes), −77 kg (2 athletes), −85 kg (2 athletes), −94 kg (4 athletes), and +105 kg (2 athletes). All athletes had a training age of >7 years, and they all competed at international championships. They were free from any musculoskeletal or neurological disease or injuries at the time of data acquisition. As members of the German national team, all athletes provided written informed consent that is part of an athlete’s contract with the German Weightlifting Federation. The study was conducted in accordance with the latest version of the Declaration of Helsinki, and the protocol was approved by the ethics advisory board of the University of Leipzig (No. 2020.01.10_eb_30).

### 2.2. Source of Data

The Research Group Weightlifting at the Institute for Applied Training Science (IAT, Leipzig, Germany) has been working in collaboration with the German Weightlifting Federation for over 30 years. Among others, researchers support coaches and athletes with biomechanical analyses during training camps and competitions. For this study, biomechanical analyses assessed during training camps were used and processed with a custom-made software entitled Realanalyzer (IAT, Leipzig, Germany) that analyzes barbell kinematics [25]. Of note, training camps which immediately precede a competition (max. three weeks before) are characterized by a standardized test session to assess maximal snatch and clean and jerk performances. These test sessions always start with an individualized warm-up program for 15–20 min (e.g., cycling on an ergometer at submaximal intensity, mobility exercises with and without the barbell). Testing started with the snatch exercise. During the snatch, all athletes tried to reach their planned maximum (100%) within 8–10 load-stages. This corresponds to 8–10 sets with 1–2 reps per set, and an overall number of 10–13 reps. The rest between sets and reps was 3–5 and 1–2 min, respectively. These tests were video captured by tracking the trajectory of the barbell using Realanalyzer software. For the purpose of this study, the database Realanalyzer was retrospectively searched to identify these training sessions and data sets that were scheduled prior to competition. Only data from athletes of the German national team who performed the snatch test were selected. Originating from the highest snatch result (100%), six additional load-stages (95%, 90%, 85%, 80%, 75%, 70%) were selected for further analyses (Table 1).

### 2.3. Characterization of the Acceleration Phase

From the retrospective data, the acceleration phase of the snatch was selected for further analyses. The acceleration phase of the snatch was divided into three sections: 1st pull, transition, and 2nd pull. In general, the 1st pull is characterized by an extension of the joints in the lower extremities until the body starts being realigned with the barbell [26]. The start of this realignment (slight forward movement of the lifter: bending the knees, forward shift of shins) occurs at the end of the 1st pull and is the beginning of the transition phase. Transition ends as soon as a second extension of the joints in the lower extremities (ankle joint, knee joint) begins, and the lifter reachesthe power position (beginning of the 2nd pull) [27]. The 2nd pull ends as the vertical barbell velocity reaches its maximum [1]. Although the movement patterns during the acceleration phase are complex and consist of multi-joint motions, the knee joint angle has frequently been used as a quantitative measure to define the sub-phases of the acceleration phase [2]. According to Knudson and Morrison [28], we used a qualitative approach to divide the single phases. The described body positions at the end of the 1st pull and the end of transition can easily be detected by observing the video clip without using quantitative data (e.g., knee joint angle). In addition, the maximal vertical barbell velocity was used to define the end of the 2nd pull. With this approach, the vertical barbell velocity was matched with every event of the three sub-phases. In a preliminary analysis, we compared this qualitative sub-phase division with a quantitative sub-phase division using 3D kinematics (i.e., knee joint angle). A nearly perfect association was found between the qualitative and the quantitative approach with r = 0.99 and a mean difference in velocity of 1.9% at the end of the 1st pull and at the end of the transition, respectively. In addition, if athletes showed a drop in barbell velocity during the transition (two velocity peaks) as previously described by Baumann, Gross, Quade, Galbierz, and Schwirtz [19] and Garhammer [4], the first velocity peak and the subsequent velocity minimum fits perfectly to the qualitatively defined end of the 1st pull and the end of the transition.

### 2.4. Barbell Kinematics

Barbell kinematics were analyzed using the software program Realanalyzer. This software operates with a dv-camcorder at 50 fps and analyzes the barbell movement by an OpenCV template matching algorithm [29]. The position of the digital camera followed a routine setup, with the height of the camera 1 m above the floor and positioned next to the athlete at a distance of 5 m. The tracking results are 2D coordinates (x, y) of the barbell that were calibrated via the diameter of the barbell plates. Raw distance-data of the barbell were smoothed with a cubic spline function. Vertical velocity-time and acceleration-time-data were calculated. All data were stored in a software-specific database to be used for further analyses. This system has proven to be highly reliable for distance (ICC > 1.00), velocity (ICC > 0.99), and acceleration parameters (ICC > 0.98) of the barbell [30].

The sub-phase division yields three vertical velocity parameters: v_1st_ (barbell velocity at the end of the 1st pull), v_trans_ (barbell velocity at the end of transition), v_2nd_ (barbell velocity at the end of the 2nd pull). Thereafter, velocity increases or decreases during each sub-phase were calculated for each athlete by computing velocity differences: Δv_1st_ (velocity difference from lift off to v_1st_), Δv_trans_ (velocity difference from v_1st_ to v_trans_), Δv_2nd_ (velocity difference from v_trans_ to v_2nd_). Finally, these velocity differences were determined for each of the seven load-stages.

### 2.5. Statistical Analyses

In a first step, a linear regression model was used to calculate the LvR slope for Δv_1st_, Δv_trans_, Δv_2nd_ with increasing barbell loads for the individual athlete. As a goodness-of-fit measure for the regression models, the standard error of the estimate (SEE) was used. The individual slopes were averaged to obtain group means for each sub-phase. With reference to the directed hypothesis, planned comparisons were computed for the analyzed acceleration sub-phases using repeated measures contrast analysis [31]. In brief, compared with omnibus testing using ANOVA, the contrast analysis tests an a priori postulated hypothesis based on contrast weights and a one-sample *t*-test [32]. Contrast-weights for Δv_1st_, Δv_trans,_ Δv_2nd_ are −2, 1, 1, respectively. Hedges *g* effect sizes were calculated from the *t*-score of the *t*-test. The above-described statistics were processed using Microsoft Excel (version 2016). In addition, 95% confidence intervals for the effect size measure was calculated in R (version 3.6.3) [33]. The significance level (one-tailed) was set to *p* ≤ 0.05. In accordance with the conventions of Cohen [34], g ≥ 0.2 indicates a small effect, g ≥ 0.5 a moderate effect, and g ≥ 0.8 a large effect.

## 3. Results

The average barbell velocities for the 1st pull, transition, and the 2nd pull are reported in Table 2. All regressions models for the LvR showed an acceptable fit with a SEE (mean ± SD) of 0.034 ± 0.012 m/s for Δv_1st_, 0.036 ± 0.014 m/s for Δv_trans_, and 0.054 ± 0.03 m/s for Δv_2nd_.

**Figure 1:**
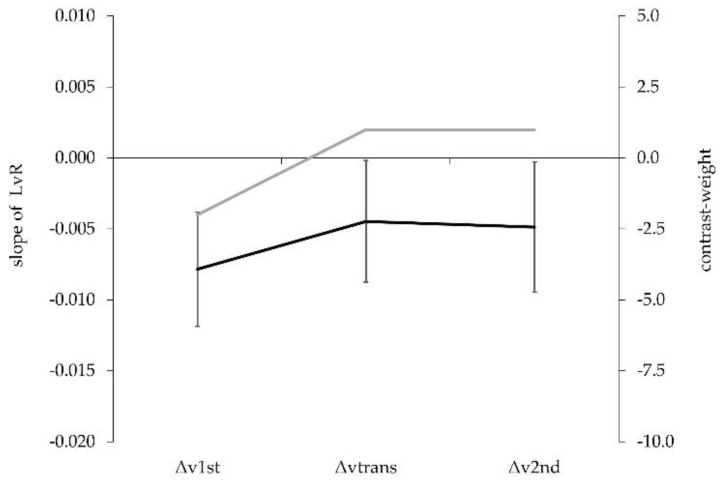
Group data (mean ± SD) illustrating the LvR slope in sub-phases of the snatch acceleration phase (black) and a priori contrast-weights of our study hypothesis (grey).

For group mean data, the increased barbell load resulted in a decreased acceleration impulse and, thus, a negative velocity difference (i.e., negative slope) for all sub-phases within the acceleration phase. The contrast analysis showed a significant effect for the a priori postulated hypothesis (*t* = 1.85, *p* = 0.044, g = 0.49 [−0.05, 1.04]). Increased loads resulted in a higher velocity loss in the 1st pull compared with the transition or the 2nd pull (Figure 1).

**Figure 2:**
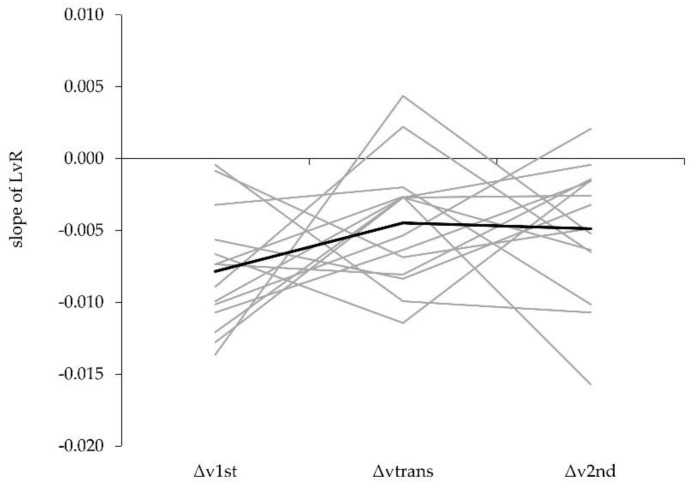
Group mean data (black) and individual responses (grey) for LvR slopes.

Although a contrast analysis has a high test-power [32], the overall statistical effect was rather low. This might be due to the large variance around the mean of the slope of the LvR of the single sub-phases (Figure 2). Because the velocity loss across the different sub-phases follows a linear function (LvR), the large variance can be rated as an expression of linear individual responses (i.e., different LvR slopes).

**Figure 3:**
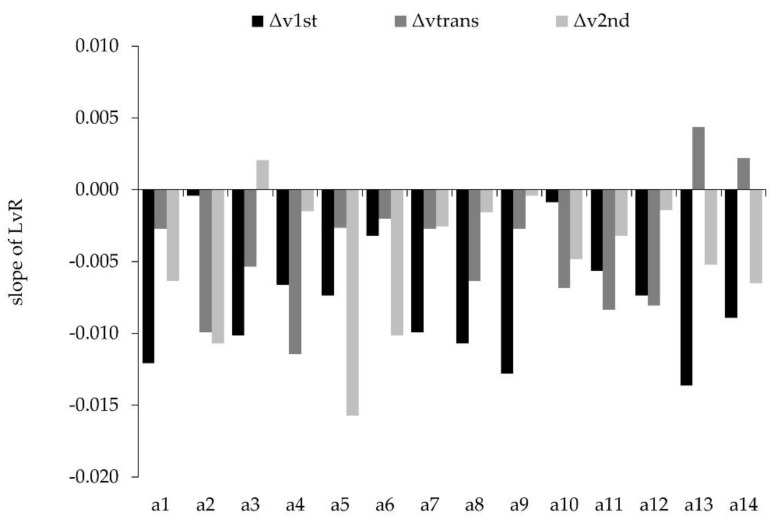
Individual LvR profiles (slope) of single athletes during the sub-phases of the snatch acceleration phase.

Accordingly, we checked the individual LvR slopes for all sub-phases and for each athlete. This additional procedure yielded highly individualized profiles (Figure 3).

**Figure 4:**
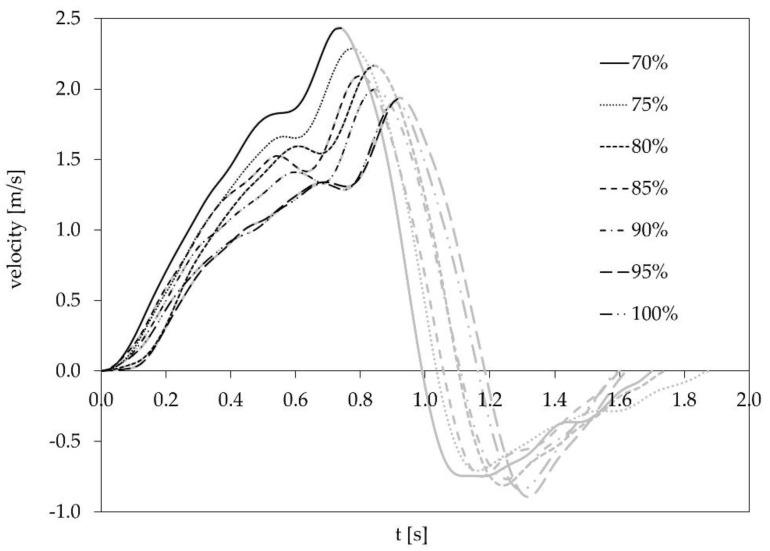
A representative example of the vertical barbell velocity during the snatch from lift off to the deep squat position for Athlete #9. The black part of the lines indicates the acceleration phase from lift off to maximum vertical barbell velocity.

According to the group mean values, our study hypothesis can be confirmed. However, the single case analysis proved that only seven athletes showed the hypothesized pattern. The remaining seven athletes had different LvR profiles. Of note, each profile corresponds to the individual effect of an increased barbell load on the vertical barbell velocity during the acceleration phase (Figure 4).

## 4. Discussion

High snatch performances can be achieved if the underlying training program is able to improve the lifter’s physical qualities. For this purpose, the training program of weightlifters consists of several specific exercises in combination with different training methods during a periodized macrocycle [13,16]. The training response should account for a higher amount of force in the single sub-phases to accelerate a higher barbell load [9]. Optimal training programs are based on the biomechanical analysis of the desired competition and training exercises (i.e., needs analysis) [35]. With reference to this approach, a LvR analysis was realized for the different sub-phases of the acceleration phase during the snatch in elite male weightlifters of the German national team. LvR slopes or force–velocity relationships are suitable means to rate athletes’ neuromuscular capabilities [36,37,38]. Previous studies have used the LvR approach to calculate a theoretical 1RM and to rate relative intensity during strength training [11,39,40]. In this study, the LvR was used to evaluate how an increased barbell load affects athletes´ accelerative capabilities within the different sub-phases of the acceleration phase during the snatch. Data from these analyses can be used for training programming. A high negative slope of LvR during a given sub-phase is the result of a high-velocity loss due to decreased acceleration capabilities of the neuromuscular system. The selection of exercises for the acceleration phase should, therefore, specifically target the sub-phase were the highest negative LvR slope is present. If accelerative capabilities in this particular phase improve, a higher vertical barbell velocity can be achieved at the end of the 2nd pull and, therefore, a higher load can be lifted. Data from the sub-phases of the acceleration phase using LvR profiles are useful for performance testing and to optimize training strategies by detecting a weak link (i.e., deficit) in the acceleration chain.

For the group mean data, the highest negative LvR slope was present during the 1st pull in comparison to the transition and the 2nd pull. These findings are in agreement with our study hypothesis. Of note, Ammar et al. [41] reported comparable results for the vertical barbell velocity in the clean from 85% to 100%. From these findings, it seems that the load-dependent velocity decrease at the end of the 2nd pull is mainly caused by the velocity decrease at the end of the 1st pull [41]. Having said this, it can be hypothesized that a specific training program is needed for the 1st pull to improve the snatch performance. In this context, it is not sufficient to simply use group mean data to monitor the training process of elite athletes [42,43]. Single case analyses are mandatory to identify individualized responses. In contrast to the postulated hypothesis, the athletes showed highly individualized LvR profiles for the sub-phases of the snatch acceleration phase. This again reinforces the need for case studies and single-subject assessment in elite athletes [44]. For example, Athlete #2 (a2) and Athlete #9 (a9) showed completely different LvR profiles, with a9 having the highest negative LvR slope in the 1st pull while a2 had the highest negative LvR slope in the transition and 2nd pull. Based on these data, it can be hypothesized that both athletes may not improve their snatch performance if they exercise according to the same training program. As a consequence of this data-driven individualized approach, we can detect athletes’ strengths and weaknesses. More specifically, if the largest loss in barbell velocity occurs in the 2nd pull, it does not make sense to focus on the 1st pull during training and vice versa. Coming back to the two aforementioned examples, a9 would benefit from an emphasized training of the 1st pull to increase snatch performance, while a2 would benefit from training the transition and the 2nd pull.

The design and development of adequate training programs to improve the acceleration phase of the snatch depend on the loading scheme and the exercise selection. In general, the 1st pull is categorized as more strength-oriented while the 2nd pull is more power-oriented [24,45]. To meet these demands, heavy snatch pulls with loads >100% of the personal best (i.e., snatch deadlifts) are suitable to train the 1st pull of the snatch [46]. Because of the short time to accelerate the barbell in the 2nd pull (ca. 0.16 s [6]) and the existing barbell velocity at the beginning of the 2nd pull, the athlete needs both a high rate of force development and a high movement velocity to further accelerate the moving barbell from an almost upright posture (i.e., power position). We hypothesized that the already existing vertical barbell velocity at the beginning of the 2nd pull is a reason why one athlete showed the unexpected outcome of a positive LvR slope. As a prerequisite for the 2nd pull, either an exercise starting from the blocks or from hang position (i.e., hang pull, midthigh pull) should be used with loads >120% to develop adequate rates of force development or loads <100% to maximize barbell/movement velocity and peak power [15,47]. In addition, snatch pulls can be used to exercise the 2nd pull because of the general overemphasis of the 2nd pull during snatch pull drills in contrast to the 2nd pull during the snatch [46]. Moreover, training of the transition phase seems less effective than training the two other sub-phases. According to Walsh [48], the transition can be seen as a by-product from the 1st pull to execute the 2nd pull through a realignment of the lifter’s body. This is in agreement with results from Bartonietz [9], as well as Böttcher and Deutscher [1], who ascribed a technical flaw during the transition to the poor execution of the 1st pull and the initial conditions for the beginning of the transition, respectively. Athletes with a high negative LvR slope during transition should, therefore, additionally focus on the execution of the 1st pull in order to optimize acceleration during the transition. Our analyses revealed two athletes with positive LvR slopes during the transition. As already described for the 2nd pull, it can be hypothesized that the pre-existing vertical barbell velocity at the beginning of the transition phase can be a reason why a positive slope may occur.

Velocity testing during the sub-phases of acceleration in weightlifting is rather easy-to-administer using commercially available or even freeware devices to track the barbell motion (e.g., linear position transducer, accelerometer, video tracking). Finally, the LvR slope can be calculated using Microsoft Excel. When using this data-driven approach, coaches have the opportunity to select specific exercises and loading schemes according to the needs of each individual athlete. When using the LvR profile for long-term performance testing over a macrocycle, coaches can track individual improvements of their athletes´ acceleration capabilities in response to training. 

This study has some limitations that warrant discussion. First, we acknowledge that the derived recommendations for the training of sub-phases are based on logical conclusions. It is not yet clarified whether performance testing using data from sub-phase LvR slopes really results in improved overall snatch performance. Second, the results of this study do not reflect a potential interaction between the sub-phases. As mentioned earlier, for example, the execution of the 1st pull has an impact on the subsequent performance during transition. Third, the linear fit of the LvR regression model for the 2nd pull was somewhat impaired compared with the 1st pull and transition. This may influence the slope, and it could have an impact on our conclusions. Accordingly, more research is needed to illustrate whether the LvR slope is an appropriate marker to detect deficits during the acceleration phase in the snatch. All limitations should be kept in mind when applying this approach in a training process with elite weightlifters.

## 5. Conclusions

The assessment of weightlifters’ sub-phase LvR profiles is an easy-to-administer approach to help coaches design individualized training programs. Findings from LvR data according to the sub-phases of the acceleration phase during the snatch help to identify athletes’ strengths and weaknesses. This information can be used to specifically tailor exercise programs according to the needs of each athlete. Finally, this study clearly shows the need to perform single-case analyses when working with elite athletes.

## Figures and Tables

**Table 1:** Average absolute barbell loads (kg) at the relative load-stages (mean ± SD).

70%	75%	80%	85%	90%	95%	100%
102.5 ± 16.3	111.1 ± 16.7	120.0 ± 18.0	128.2 ± 19.9	135.0 ± 20.7	144.1 ± 21.8	149.4 ± 22.0

**Table 2:** Group data of vertical barbell velocity (m/s) for the sub-phases of the acceleration phase (mean ± SD).

Sub-Phase	70%	75%	80%	85%	90%	95%	100%
1st pull	1.41 ± 0.19	1.35 ± 0.18	1.31 ± 0.18	1.27 ± 0.18	1.23 ± 0.21	1.19 ± 0.2	1.17 ± 0.17
transition	1.70 ± 0.18	1.63 ± 0.18	1.55 ± 0.14	1.48 ± 0.14	1.42 ± 0.15	1.37 ± 0.13	1.34 ± 0.11
2nd pull	2.40 ± 0.11	2.27 ± 0.08	2.20 ± 0.09	2.11 ± 0.10	2.02 ± 0.09	1.94 ± 0.08	1.89 ± 0.07
